# Knee resurfacing with double unicompartimental arthroplasty: rationale, biomechanics, indications, surgical technique and outcomes

**DOI:** 10.1186/s40634-021-00402-6

**Published:** 2021-09-15

**Authors:** Sergio Romagnoli, Stefano Petrillo, Matteo Marullo

**Affiliations:** Department of Joint Replacement, IRCCS Istituto Ortopedico Galeazzi, via R. Galeazzi 4, 20161 Milan, Italy

## Introduction

Nowadays, there are few data available about rationale, indications and outcomes of bi-unicompartimental knee arthroplasty (bi-UKA). Despite the continuous progress and innovation in surgical techniques and prosthetic materials, there is still a trend to manage patients with severe bi-compartmental femoro-tibial knee osteoarthritis (OA) and healthy anterior cruciate ligament (ACL) using a total knee arthroplasty (TKA).

It is well known that TKA is a safe and effective surgical option in case of tri-compartmental knee OA, with associated reduced pain and improved function, and with a survivorship of more than 90% at 10 years of follow-up [12, 14, 18]. These results have allowed to consider TKA as the gold standard in the treatment of patients with severe tri-compartmental knee OA.

On the other hand, when isolated OA of the medial or lateral femoro-tibial compartment of the knee is present and associated with a biomechanically functional ACL, a good surgical option with excellent clinical and functional results could be a unicompartimental knee arthroplasty (UKA). Several studies have demonstrated that the key of success and feasibility of UKA is the integrity of the ACL [1, 13, 23]. As for TKA, the survivorship of UKA at 10 years is approximately 85–90%, and the main cause of failure is the progression of OA in the contralateral compartment [4].

Many advantages were associated with the use of UKA over TKA: isolated replacement of only one knee compartment, soft tissues sparing and bone stock preservation, ACL maintaining with consequent good proprioception, and easily revision. Furthermore, a reduced blood loss, a more rapid recovery, a reduced hospital length of stay and excellent range of motion (ROM) were reported in several studies [1, 6, 9, 15, 16].

Then, following these results, UKA is becoming the gold standard for the management of isolated compartmental knee OA.

The more challenging problem in knee replacement surgery rises up in patients with combined medial and lateral OA and healthy ACL. In this kind of patients, according to our experience, a bi-UKA should be considered [19, 20]. ACL integrity is crucial to reach excellent outcomes. Indeed, the ACL is fundamental for the knee kinematics, preserving the femoral roll-back and the screw-home mechanics (external rotation of the tibia in fully knee extension) [11].

Thus, by preserving the ACL, the natural knee kinematics remains close to the native one, even if a UKA or a bi-UKA is implanted [1].

The importance of the ACL in knee kinematic was known since the 1970s, when several ACL-retaining TKAs were developed. These implants were essentially made of two connected medial and lateral unicondylar implants and preserved the tibial eminentia and the cruciate ligaments. The earliest and most famous were the polycentric knee, developed by Gunston; the Marmor modular knee, designed by Marmor; and the Geomedic, developed at the Mayo Clinic [8, 10, 17]. These ACL-retaining TKAs demonstrate a kinematics closer to normal than ACL-sacrificing TKAs, and knees are reported to feel more normal. But they were technically difficult to implant and lacked adequate instrumentation. Knee flexion was often restricted, and mechanical loosening occurred frequently. These problems, united with the progressive improvement of clinical results and survivorship of ACL-sacrificing TKAs, led to a progressive abandon of ACL-retention TKAs [2, 7].

The aim of this study is to report the rationale, biomechanics, indications, surgical technique and outcomes of bi-UKA in patients with associated medial and lateral OA and with a biomechanically healthy ACL. This study could encourage the use of bi-UKA respect to TKA, especially in young high demands sports patients.

## Biomechanics

From a biomechanical point of view, bi-UKA restores a knee kinematics closer to the native one respect to TKA. The femoral condyles roll-back and the screw-home mechanics of the tibia are preserved only if the ACL is maintained, as in UKA or bi-UKA implants [1, 11].

Stiehl et al. have shown with bi-planar fluoroscopy that TKA, using both posterior stabilized or cruciate retaining liners, determines a posterior dislocation of the femur in fully knee extension [24]. Moreover, an anterior sliding of the femur from 0 to 30 degrees of knee flexion was observed. These results have highlighted the “paradoxical” motion determined by TKA, which can be avoided only preserving the ACL.

In 2005 we published a pioneering study that compared knee kinematics in patients with well-functioning cruciate-preserving medial UKA and bi-UKA [1]. Seven patients with a medial UKAs and five with bi-UKAs were enrolled in the study. Evaluation was performed using dynamic fluoroscopy during treadmill gait at 1 m/s, single limb stepping up and down, maximum flexion kneeling on a padded stool, and weight- bearing straight-leg stance.

Both groups showed less than 2 mm of posterior translation of the medial condyle during kneeling. Posterior translation of the lateral condyle in the bi-UKAs averaged 4 mm during kneeling activities. Both groups showed tibial internal rotation with flexion during the stair activity. For 0–30 degrees flexion during stair activity, the medial condyle translated posterior 3.5 ± 2.5 mm in UKAs and 4.7 ± 1.9 mm in bi-UKAs (*P* = 0.035). Lateral condyle posterior translation was 5.0 ± 2.3 mm in bi-UKAs for 0–30 degrees of flexion. During gait, the bi-UKAs showed greater knee flexion from heel strike to midstance phase than UKAs (*P* < 0.01), but similar flexion from late stance through swing phase. The bi-UKAs showed greater tibial external rotation throughout stance phase (*P* < 0.01), which correlates closely to greater posterior translation of the medial condyle in early to midstance phase (*P* < 0.01) This preliminary study demonstrated that retaining the ACL maintains some basic features of normal knee kinematic like posterior translation of the condyles and tibial internal rotation during flexion (roll-back movement). However, it did not compare directly bi-UKA to TKA or to healthy knees. So, recently we performed a gait-analysis study enrolling 9 patients: 6 with bicompartmental knee OA and intact ACL in a knee and an intact contralateral knee, and 3 with healthy knees [22]. Three patients with OA received a bi-UKA while the other 3 patients had a mobile-bearing TKA. The gait analysis evaluation was performed preoperatively and 6 months postoperatively. The main findings of this study were: stance and swing percentage values of bi-UKA patients were more similar to the healthy subjects than TKA patients; the number of steps per minute accomplished by bi-UKA patients were only 7% lower than healthy subject value, while for TKA patients it was 20% lower; step length was 15% and 30% lower than healthy subject for Bi-UKA and TKA, respectively. Evaluating knee flexion-extension during the gait circle, bi-UKA perfectly reproduced the normal gait circle, while TKA patients did not properly extend the operated knee during the stance phase and did not flex adequately the knee during the swing phase. Consequently, TKA patients excessively abduced the hip during the whole swing phase compared to Bi-UKA patients. Even if this was only a preliminary study with a limited number of subject enrolled, it showed that bi-UKA is superior to TKA in reproducing a gait pattern closer to the one of healthy knees.

## Indications for bi-UKA

The indications for a bi-UKA are numerous, but the real issue of such procedure is the selection of the patients and a careful assessment of ACL integrity. An accurate clinical and functional evaluation are fundamental to investigate patient’s symptoms, demands, preoperative daily-life activities and postoperative expectations [19, 22].

The radiographic investigation of the knee including anteroposterior, lateral, Merchant and Rosenberg views are crucial to reach definitive indications. The magnetic resonance imaging study of the knee is indicated in patients with a suspected ACL deficiency or patello-femoral pain.

The ideal candidate for a bi-UKA is an individual younger than 60 years, involved in high demands sports, in particular men. We used to consider bi-UKA in patients with combined severe medial and lateral femoro-tibial OA (at least Kellgren-Lawrence grade II) and both healthy patellofemoral joint (or no symptoms) and ACL; knee ROM major than 90 degrees; flexion contracture minor than 5 degrees, axial malalignment lower than 10 degrees, which can be passively corrected; and tibial bony defect minor than 12 mm [19, 22].

**Fig. 1:**
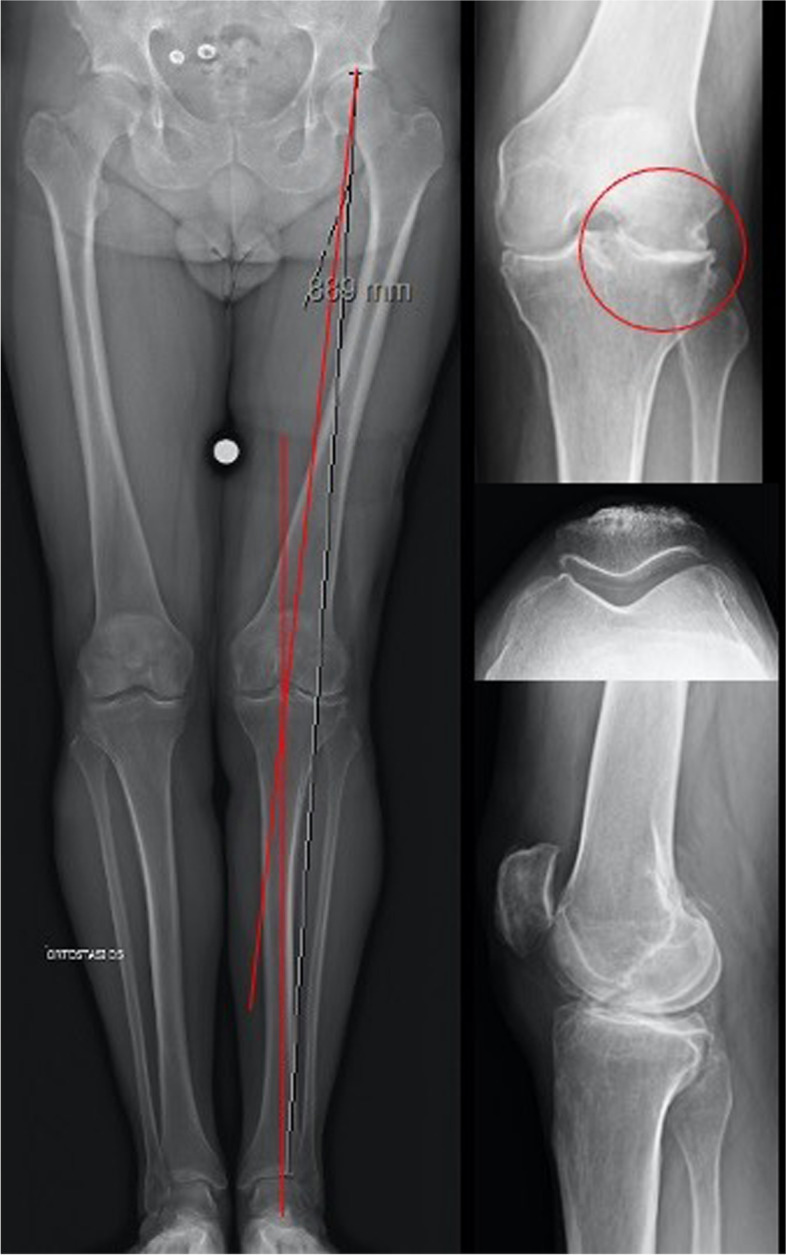
Preoperative X-rays evaluation of a 56 years-old male with a native varus morphotype, who underwent a lateral open meniscectomy 15 years before in his left knee. Consequently, he developed a grade IV lateral OA, a grade III medial OA and a valgus alignment

**Fig. 2:**
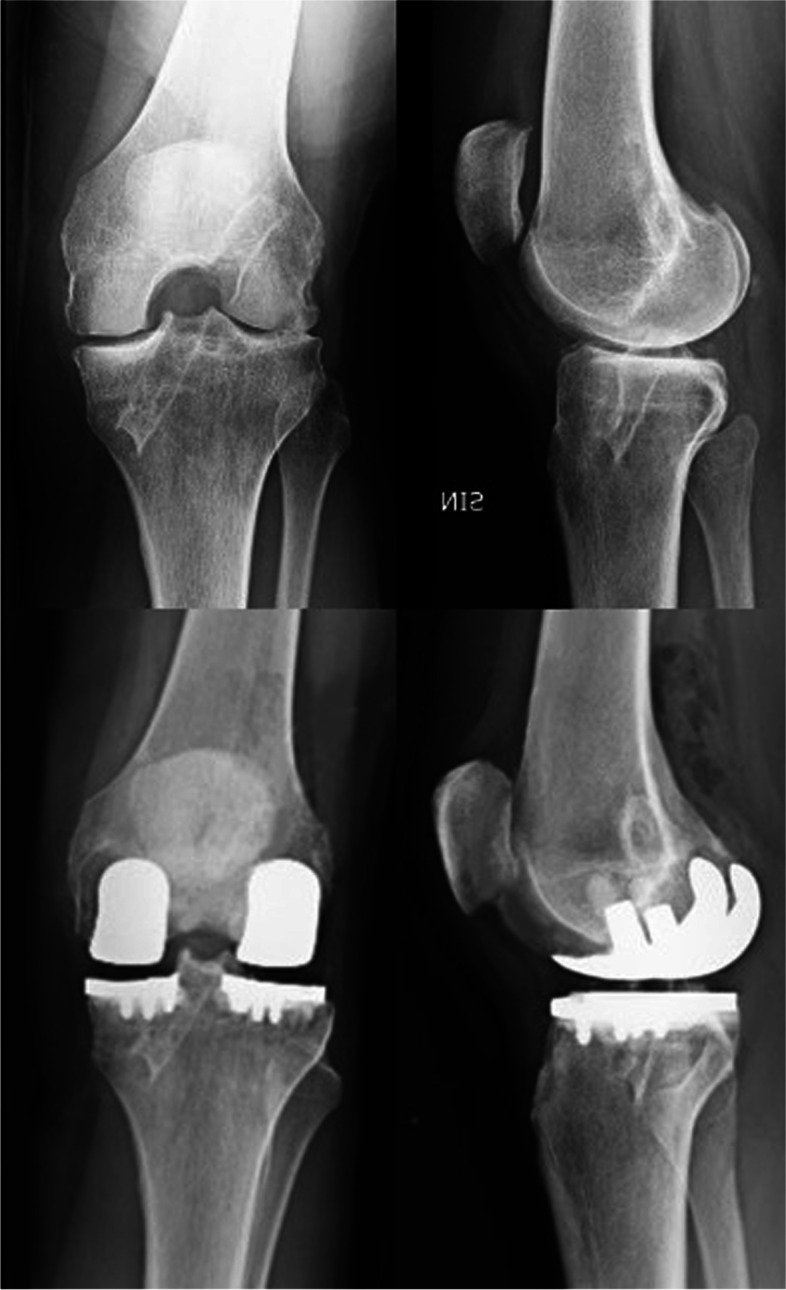
Fifty-eight female patient who had ACL reconstruction with patellar tendon 10 years before and presented tibiofemoral OA. The ACL was anatomical and well-functioning, so a Bi-UKA was performed

Other than patients with bi-compartmental femoro-tibial OA, even patients with a valgus or a varus knee who underwent medial or lateral meniscectomy respectively could benefit from a bi-UKA (Fig. 1). Moreover, osteonecrosis of both medial and lateral femoral condyle or tibia represent another indication for bi-UKA. Patients with a previous ACL reconstruction who developed combined medial and lateral femoro-tibial OA can represent another indication, only if the ACL is biomechanically functional (Fig. 2). Finally, bi-UKA could be performed in UKA revisions for OA progression in the contralateral compartment. If the previous UKA has not signs of loosening or polyethylene wear and the ACL is still functional, implanting another UKA in the contralateral, damaged compartment results in a Bi-UKA, a less invasive solution than revising the UKA with a TKA [21].

On the other hand, as for UKA, bi-UKA should be contraindicated: in patients with chronic inflammatory diseases, patellofemoral symptoms or malalignment; knee instability; severe coronal or sagittal deformity; and tibial bony defect major than 12 mm [19].

## Surgical technique

The surgical technique of bi-UKA is the same surgical technique as UKA applied to both the medial and lateral compartments. All our knee arthroplasty procedures are performed without using tourniquet. Two different surgical approaches can be used: a medial para-patellar or a double mini skin incision (one medial of 4–6 cm and another lateral of 6–8 cm). We use this second approach in patients who had previous skin incisions, as in revision surgery, osteotomy or fracture sequelae cases; anyhow, particular attention should be taken to respect the supero-medial geniculate artery to ensure adequate patellar blood supply.

Our standard approach consists of a minimally invasive medial para-patellar skin incision of 8–10 cm and a mini-midvastus arthrotomy; in this way it is possible to easily expose both medial and lateral femoro-tibial compartment of the knee, without patellar eversion.

The surgical procedure continues like performing two consecutive UKAs. We suggest starting from the medial compartment in varus knees, and lateral compartment in valgus knees. In this manner, the correction of the coronal deformity could be easily achieved, albeit maintaining the native knee coronal alignment. After correction of the deformity by implant trial positioning, the other compartment is addressed.

**Fig. 3:**
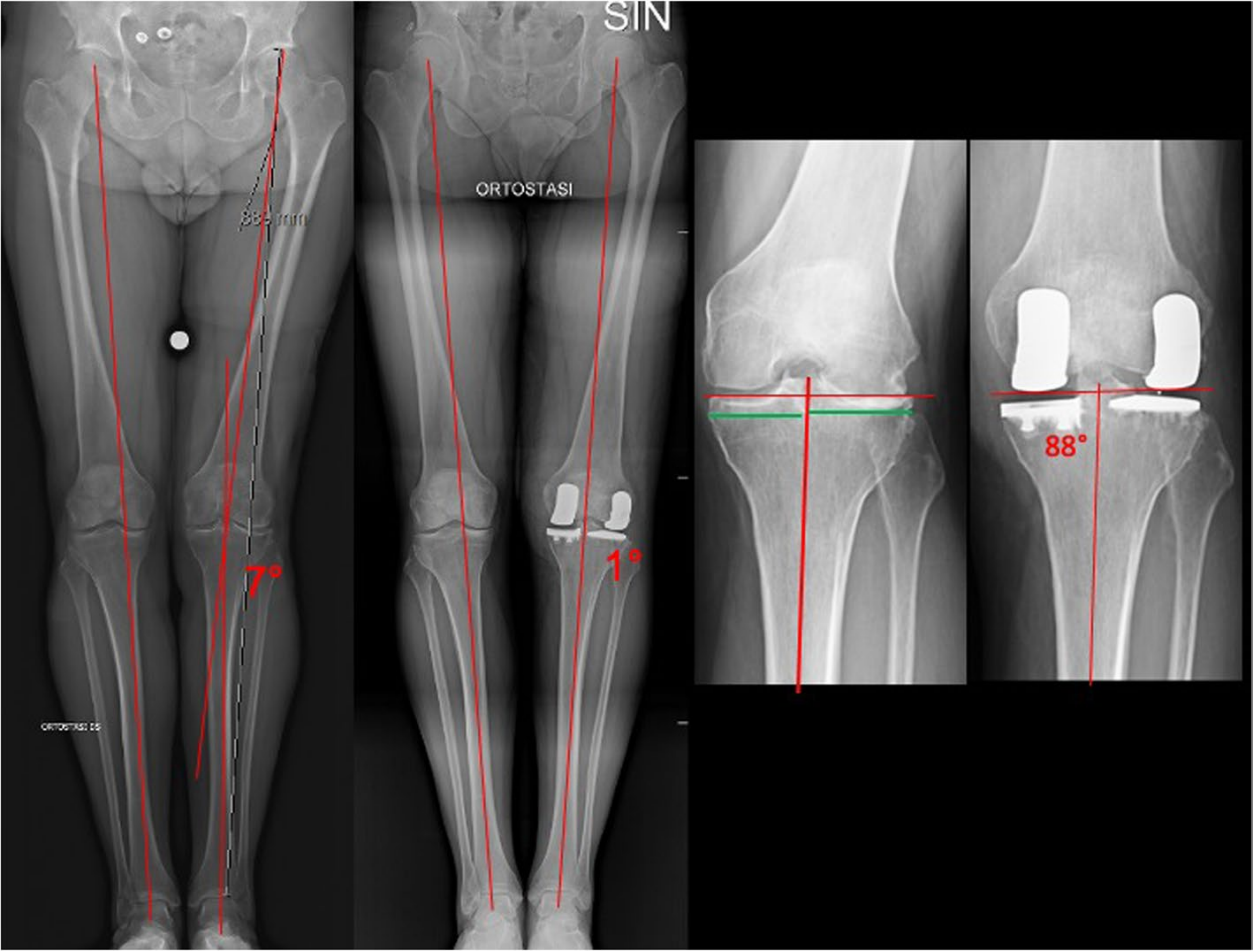
Respect of the morphotype. Same case of Fig. 1. The native coronal alignment of the patient was in varus, so the preoperative planning was performed with the aim to restore the native morphotype

We prefer a tibia-first technique, using a minimally invasive tibial guide at the same time in the two femoro-tibial compartments. This phase is crucial to obtain a correct coronal alignment, which should be anatomical and not mechanical. To reach this result, an undercorrection of the coronal deformity is necessary. Indeed, coronal orientation of the tibial cuts must be perpendicular to the epiphyseal axis of the tibia, respecting height and obliquity of the joint line and avoiding the necessity of any subsequent release, maintaining the native alignment of the patient (varus or valgus) (Fig. 3) [22]. This is a paramount feature of Bi-UKA. In healthy individuals, the joint line is not perpendicular to the tibial axis, but it averaged 3 degrees of varus alignment [3]. Moreover, 32% of male and 17% of female Caucasian individuals present a constitutional varus alignment major than 3 degrees, and these percentages increase in the Asian population [3].

During gait the lower limb comes into slight adduction; consequently, the joint line comes parallel to the ground, an optimal orientation for load bearing. This situation could be maintained unaltered only with UKA or bi-UKA, by preserving the ACL and undercorrecting the coronal deformity, respecting the native obliquity and height of the knee joint line [5, 22].

**Fig. 4:**
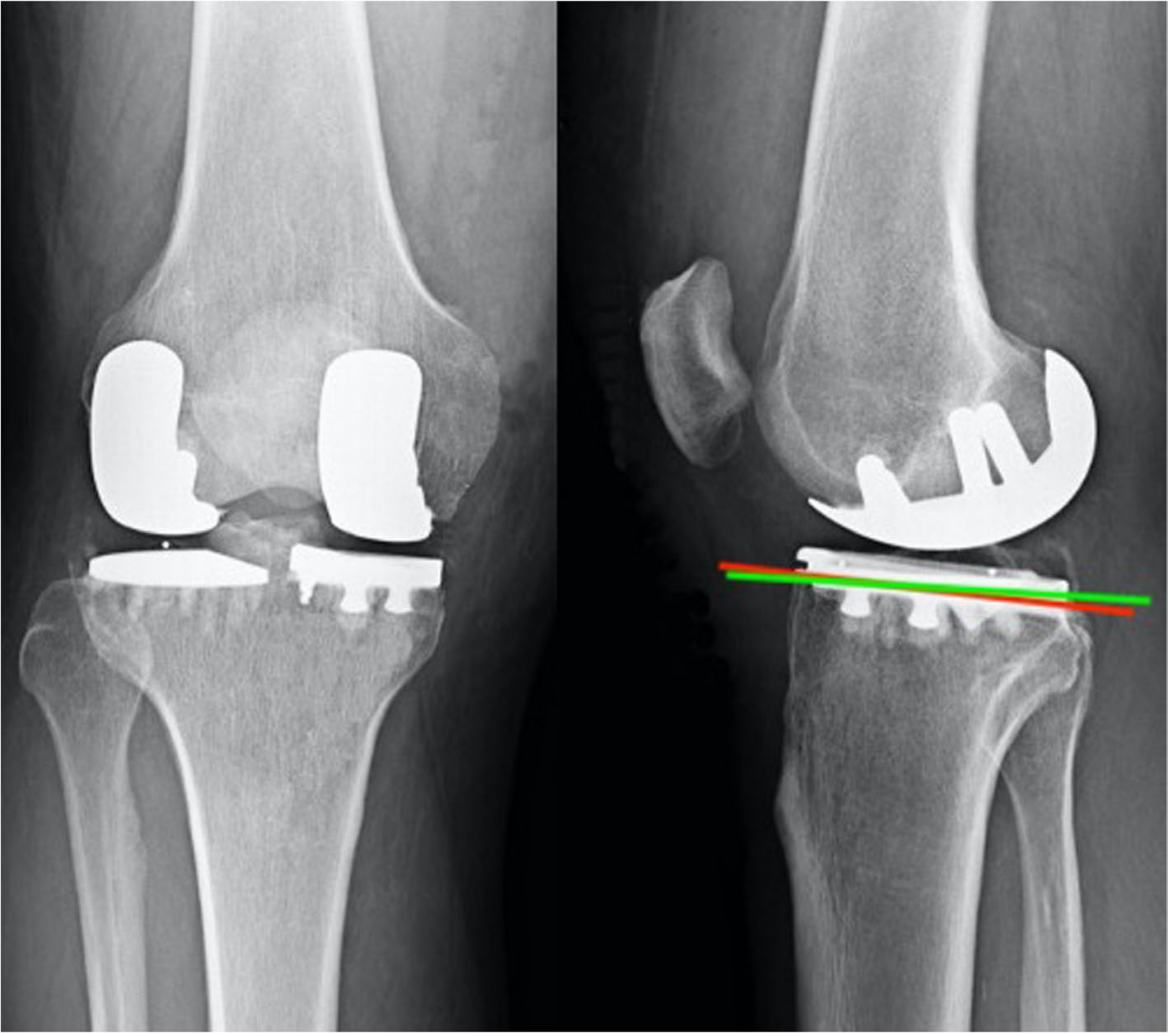
The medial and lateral compartment have different tibial slope. The medial tibial slope (red line) is steeper than the lateral one (green line)

On the other hand, in the sagittal plane, the tibial slope should be from 3 to 6 degrees in the medial side and from 0 to 3 degrees in the lateral side (Fig. 4). Reproducing the native tibial slope is essential for preserving both ACL and posterior cruciate ligament function, and consequent knee stability.

**Fig. 5:**
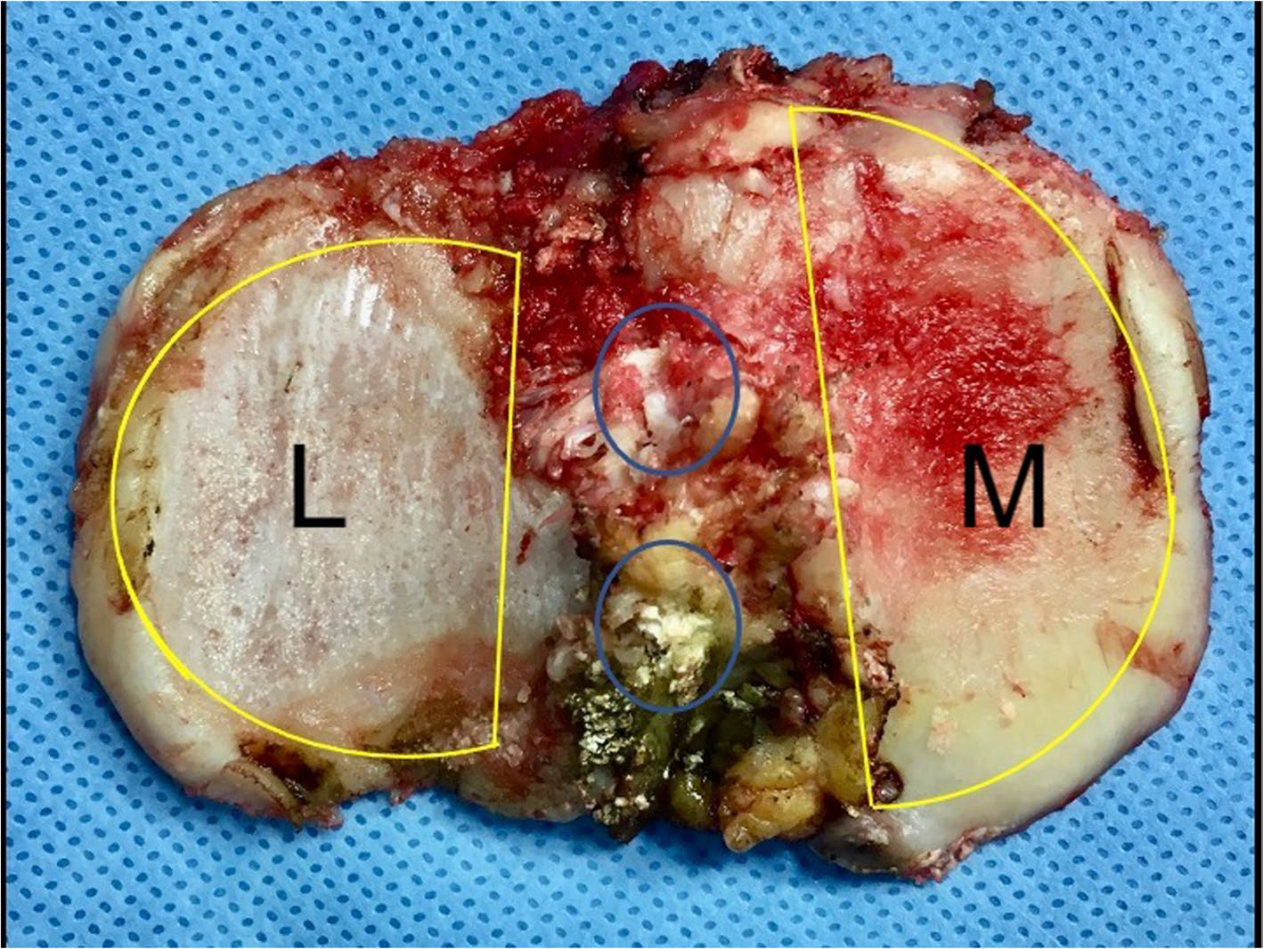
The two tibial hemi-plateaus have different shapes. The lateral one (L) is more round, the medial one (M) has a drop-like shape

An important feature to point out is that the two tibial plateaus have different shapes: more round the lateral one, drop-shaped the medial one (Fig. 5). Consequently, they should be replaced by different tibial component in order to improve bone coverage.

At this point, the varus-valgus knee stability in fully knee extension is tested. Moreover, a mark in both medial and lateral femoral condyles at the same level of the anterior border of the tibia is performed and used as reference for correct positioning of the femoral tray.

Then, using appropriate instrumentation, the distal and posterior femoral cut are performed in knee extension and flexion respectively. Cutting should be done using two different tensor guides that ensure the same amount of bone resection, to obtain a balanced flexion-extension gap.

According to the concept of “resurfacing”, only 2–3 mm of bone and cartilage should be removed. This amount of tissue corresponds to the thickness of the femoral component. Finally, the femoral component should be positioned as much lateral as possible, in both medial and lateral compartment. The tibia externally rotates during complete extension, and this trick allows the femoral components to remain perpendicular to the tibial plateau throughout all the arc of motion, both in extension and in flexion.

**Fig. 6:**
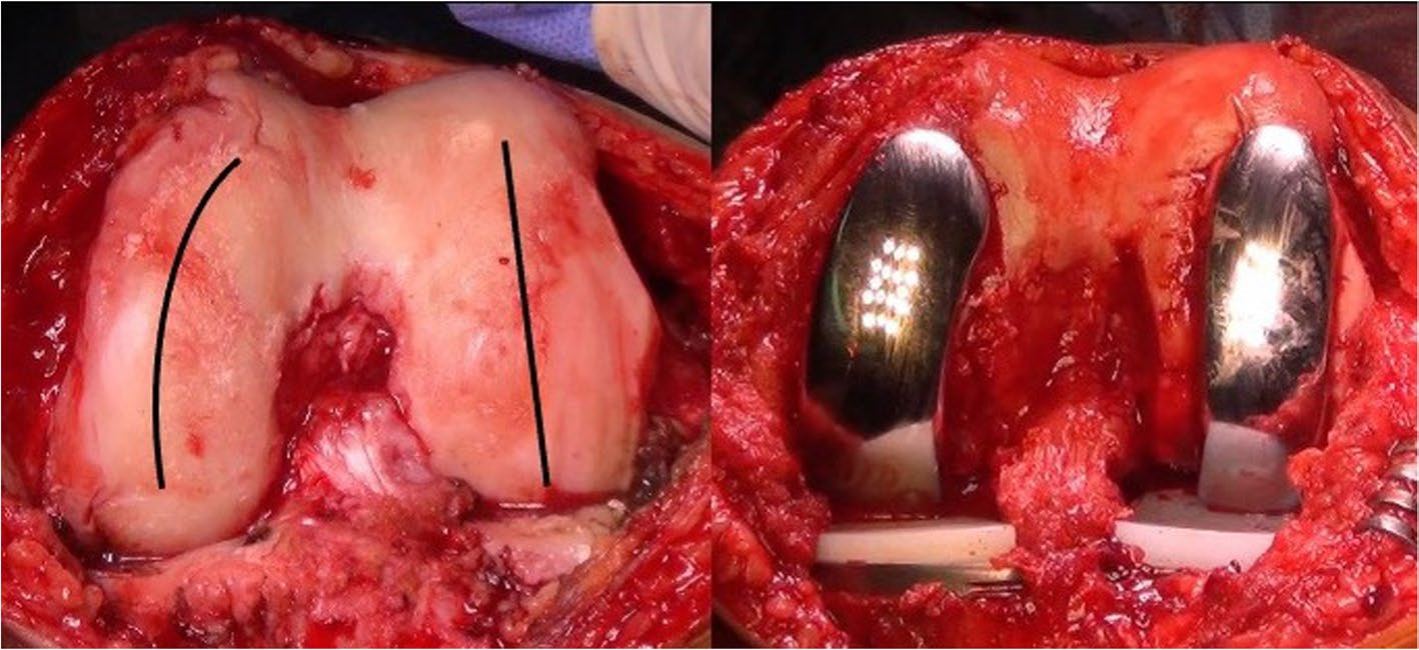
The two femoral condyles have different shapes. The lateral one is straight, the medial one is curved. Consequently, the two femoral components should have different, dedicated shapes

As the femoral condyles have different shape, straight the lateral one and more curved the medial one, we suggest using different type of femoral components: a straight one for the lateral and an anatomical one for the medial condyle (Fig. 6).

A great advantage of the bi-UKA is the possibility to choose sizes, shapes and positions of the two femoral components independently, thus allowing a close fit of the UKAs to the native knee; Bi-UKA is a real “custom-made” knee replacement.

**Fig. 7:**
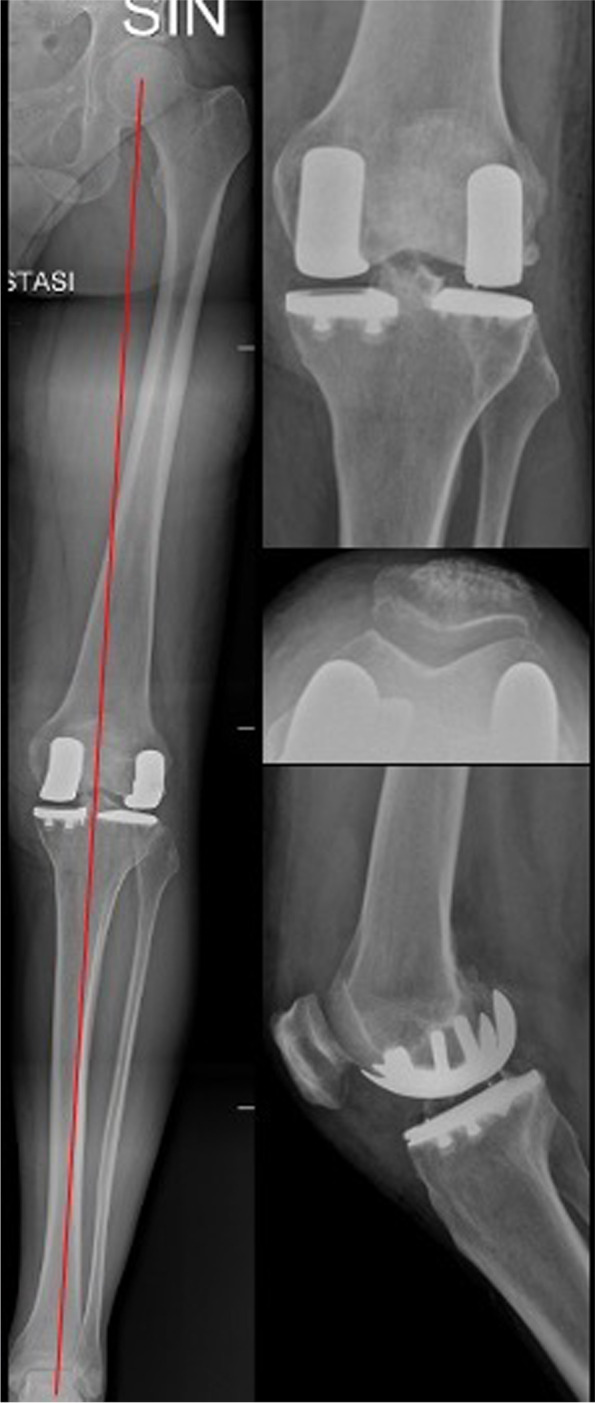
Postoperative X-rays evaluation of the case in Fig. 1. It is possible to see the respect of the native morphotype and the different prosthetic components used in the medial and in the lateral compartments

The surgical procedure ends when the trials are positioned and components, stability, ROM, and knee alignment are checked. Cementing starts from the lateral tibial side (more difficult to expose), proceeds with the medial one, and ends with the femoral components (Fig. 7).

Patients began progressive weight bearing the day after surgery. Passive and active ROM is initiated within 24 h of surgery. Patients are typically discharged from the Orthopaedic Department on postoperative day 2, after demonstrating the ability to ambulate alone with the aid of crutches and flex the knee at least 90°.

## Outcomes

The medical records of patients who had undergone primary bi-unicompartimental knee replacement at our institution between 2001 and 2019 were reviewed. Inclusion criteria were: bi-UKA performed as primary replacement, availability of complete pre- and postoperative X-rays, completeness of patients’ medical records, postoperative follow-up of at least 2 years. In the study period, 106 BKAs were performed. One patient died and 2 were lost before the end of the minimum 2-year follow-up period, leaving 103 BKAs performed in 101 patients.

Patients’ records were examined for the following variables: gender, age at surgery, body-mass index (BMI, *weight in kilograms* divided by the square of the height in meters), preoperative and postoperative knee ROM and pain measured using a visual analogue scale (VAS). Preoperative and postoperative clinical and functional evaluation was done with the Knee Society clinical and functional rating system. Knee Society scores were calculated from routine examinations performed preoperatively, at 6 and 12 months postoperatively, and yearly thereafter. Patient satisfaction with the procedure was assessed at the last follow-up and classified as: very satisfied, satisfied, the same, dissatisfied, very dissatisfied.

Preoperative and postoperative radiographic evaluation included a full-leg standing radiograph, a standing posterior-anterior radiograph of both knees at 45° of knee flexion (Rosenberg view), a true lateral view, and a 30° patellar axial view.

Revision of the prosthesis or replacement of any other compartment were considered as failure of the implant.

The patient population consisted of 69 females and 32 males with an average age at surgery of 65.2 ± 11.4 years (range: 30–86 years). Mean BMI was 26.9 ± 4.6 (18.7–36.0).

Nine patients had a previous intraarticular fracture, 3 patients previously underwent high tibial osteotomy (HTO), 3 patients had a previous ACL reconstruction, 4 had osteonecrosis of both femoral condyles, 4 had poliomyelitis with adequate muscle control.

The mean follow-up was 9.4 ± 5 years (range, 2–20). Overall, statistically significant improvements (*p* < 0.01) were observed for knee joint ROM (108.3° vs 125.4°), clinical KSS (67.7 vs 91.0), functional KSS (58.2 vs 88.4) and VAS pain score (from a median of 8 to 1).

The average preoperative coronal deformity (Hip-Knee-Ankle angle) ranged from 12° varus to 7° of valgus. The average postoperative alignment was 2,2° valgus HKA (range: from 3° varus to 2° valgus). No loosening or subsidence of the implants or signs of osteolysis were recorded.

At the final follow-up, 74 patients (73.2%) were very satisfied with the procedure, 21 patients (20%) were satisfied, and six patients (5.8%) dissatisfied.

At the mean 9.4 years of follow-up, six knees required further surgeries, yielding an overall survival rate of 94.2%. Two knees showed OA progression in the patellofemoral compartment; consequently, a patellofemoral arthroplasty was added 4.1 years and 10.3 years after the index operation. One knee showed medial polyethylene (PE) wear 16.7 years after the index operation; as the tibial component and the PE were monolithic, both the tibial component than the PE were revised with others, leaving the other prosthetic components in place. One patient had a fracture of the medial tibial plateau after a fall 3.2 years after the index operation and another patient experienced anteroposterior instability consequently to lesion of the anterior cruciate ligament 13.2 years after surgery; both patients were revised with a mobile-bearing primary TKA. One patient with poliomyelitis in the operated limb experienced antero-posterior instability 8.8 years after surgery, so the bi-UKA was revised with a hinged TKA.

## Conclusions

Bi-UKA replace the two tibiofemoral compartments, leaving the patellofemoral one and the cruciate ligaments in place, permitting to maintain many essential features of native knee kinematics, like femoral rollback and the screw-home mechanism. It is a kind of personalized knee replacement that allow to choose size, shape and orientation of the prosthetic components independently. Bi-UKA must be implanted with an anatomic alignment to give the correct tension to the cruciate ligament throughout the range of movement.

Bicompartmental replacement showed excellent clinical results, with a gait pattern and knee function closer to the native one compared to TKA and survivorship not inferior to the one of TKA.

The aim of knee replacement should be recreating normal knee kinematics and function. Bi-UKA could be a way to reach this objective.

## Abbreviations

Bi-UKA: Bi-unicompartimental knee arthroplasty
OA: Osteoarthritis
ACL: Anterior cruciate ligament
TKA: Total knee arthroplasty
UKA: Unicompartimental knee arthroplasty
ROM: Range of motion
BMI: Body Mass Index
VAS: Visual Analogue Scale
KSS: Knee Society Score
HTO: High tibial osteotomy
PE: Polyethylene

## References

[1] Banks SA, Frely BJ, Boniforti F, Reischmidt C, Romagnoli S (2005). Comparing in vivo kinematics of unicondylar and bi-uni-condylar knee replacement. Knee Surg Sports Traumatol Arthrosc.

[2] Bellemans J, Scuderi GR, Tria AJ (2010). Bicruciate-substituting and bicruciate- replacing arthroplasty of the knee: technique and results. The knee: a comprehensive review.

[3] Bellemans J, Colyn W, Vandenneucker H, Victor J (2012). The Chitranjan Ranawat award: is neutral mechanical alignment normal for all patients? The concept of constitutional varus. Clin Orthop Relat Res.

[4] Berger RA, Meneghini RM, Jacobs JJ, Sheinkop MB, Della Valle CJ, Rosenberg AG, Galante JO (2005). Results of unicompartmental knee arthroplasty at a minimum of ten years of follow-up. J Bone Joint Surg Am.

[5] Blakeney WG, Vendittoli PA, Rivière C, Vendittoli PA (2020). Restricted kinematic alignment: the ideal compromise?. Personalized hip and knee joint replacement.

[6] Brown NM, Sheth NP, Davis K, Berend ME, Lombardi AV, Berend KR, Della Valle CJ (2012). Total knee arthroplasty has higher postoperative morbidity than unicompartmental knee arthroplasty: a multicenter analysis. J Arthroplast.

[7] Buechel FF, Pappas MJ (1990). Long-term survivorship analysis of cruciate-sparing versus cruciate-sacrificing knee prostheses using meniscal bearings. Clin Orthop Relat Res.

[8] Coventry MB (1979). Two-part knee arthroplasty: evolution and present status. Clin Orthop.

[9] Drager J, Hart A, Khalil JA, Zukor DJ, Bergeron SG, Antoniou J (2016). Shorter hospital stay and lower 30-day readmission after unicondylar knee arthroplasty compared to Total knee arthroplasty. J Arthroplast.

[10] Gunston FH (1971). Polycentric knee arthroplasty. Prosthetic simulation of normal knee movement. J Bone Joint Surg Br.

[11] Johnson AJ, Howell SM, Costa CR, Mont MA (2013). The ACL in the arthritic knee: how often is it present and can preoperative tests predict its presence?. Clin Orthop Relat Res.

[12] Kim YH, Park JW, Kim JS, Kulkarni SS, Kim YH (2014). Long-term clinical outcomes and survivorship of press-fit condylar sigma fixed-bearing and mobile- bearing total knee prostheses in the same patients. J Bone Joint Surg Am.

[13] Komistek RD, Allain J, Anderson DT, Dennis DA, Goutallier D (2002) In vivo kinematics for subjects with and without an anterior cruciate ligament. Clin Orthop Relat Res (404):315–325. 10.1097/00003086-200211000-00047 PMID: 12439275 10.1097/00003086-200211000-00047 12439275

[14] Koskinen E, Eskelinen A, Paavolainen P, Pulkkinen P, Remes V (2008). Comparison of survival and cost- effectiveness between unicondylar arthroplasty and total knee arthroplasty in patients with primary osteoarthritis: a follow-up study of 50,493 knee replacements from the Finnish Arthroplasty Register. Acta Orthop.

[15] Laurencin CT, Zelicof SB, Scott RD, Ewald FC (1991). Unicompartmental versus total knee arthroplasty in the same patient. A comparative study. Clin Orthop Relat Res.

[16] Lombardi AV, Berend KR, Walter CA, Aziz-Jacobo J, Cheney NA (2009). Is recovery faster for mobile-bearing unicompartmental than total knee arthroplasty?. Clin Orthop Relat Res.

[17] Marmor L (1973). The modular knee. Clin Orthop Relat Res.

[18] Ritter MA, Berend ME, Meding JB, Keating EM, Faris PM, Crites BM (2001). Long-term follow-up of anatomic graduated components posterior cruciate-retaining total knee replacement. Clin Orthop Relat Res.

[19] Romagnoli S, Marullo M, Massaro M, Rustemi E, D’Amario F, Corbella M (2015). Bi-unicompartmental and combined uni plus patellofemoral replacement: indica- tions and surgical technique. Joints.

[20] Romagnoli S, Verde F, Corbella M, Zacchetti S, Confalonieri N, Romagnoli S (2013). The bi-unicompartmental knee prosthesis. Small implants in knee reconstructions.

[21] Romagnoli S, Verde F, Zacchetti S, Confalonieri N, Romagnoli S (2013). Revision of UKR. Small implants in knee reconstructions.

[22] Romagnoli S, Marullo M, Stucovitz E, Verde F, Corbella M, Scuderi G, Tria A (2016). Bi-unicompartmental knee protheses. Minimally invasive surgery in orthopedics.

[23] Springer B, Waldstein W, Bechler U, Jungwirth-Weinberger A, Windhager R, Boettner F (2021). The functional status of the ACL in varus OA of the knee: the association with varus deformity and coronal tibiofemoral subluxation. J Arthroplast.

[24] Stiehl JB, Dennis DA, Komistek RD, Crane HS (1999). In vivo determination of condylar lift-off and screw-home in a mobile-bearing total knee arthroplasty. J Arthroplast.

